# Adjuvant radiation therapy for older women with early-stage breast cancer: a propensity-matched SEER analysis

**DOI:** 10.1007/s12094-022-02967-9

**Published:** 2022-10-13

**Authors:** Nisha Wu, Qiao Tan, Xiaohan Su, Yewei Yuan, Lingmi Hou, Junyan Li

**Affiliations:** 1 Department of Breast Surgery, Sichuan Provincial Maternity and Child Health Care Hospital, No. 290 West Second Street, Shayan Road, Chengdu, 610031 Sichuan China; 2 Department of Clinical Laboratory, The Fifth Affiliated Hospital, Southern Medical University, Guangzhou, Guangdong China; 3 Department of Biological Targeting Laboratory of Breast Cancer, Academician (Expert) Workstation, Breast and Thyroid Surgery, Affiliated Hospital of North Sichuan Medical College, Nanchong, Sichuan China; 4 Department of General Surgery, Yingshan Hospital, Southwest Hospital of Sichuan University, 1# Maoyuan Road South, Shunqing District, Nanchong, 637000 Sichuan China

**Keywords:** Breast conserving surgery, Mastectomy radiotherapy, SEER, Elderly woman

## Abstract

**Introduction:**

The purpose was to evaluate the effect of adjuvant radiation therapy on the survival prognosis of older women with early-stage breast cancer under different surgical treatments.

**Methods:**

We collected patients from the Surveillance, Epidemiology and End Results (SEER) database. Elderly female patients (≥ 70 years) with stage I–IIB diagnosed with invasive carcinoma in 1988–2017 were included. After propensity score matching (PSM), the prognosis of patients who underwent breast-conserving surgery or mastectomy was calculated separately. The effects of radiotherapy on the survival of three special population groups (breast-conserving surgery + T1N0M0 + ER positive, mastectomy + T3N0M0 and mastectomy + T1-2N1M0) were analyzed selectively.

**Results:**

Of 106,553 older women with early-stage breast cancer were identified. 48,630 patients had received radiotherapy, while 57,923 patients had not. After PSM, older women undergoing breast-conserving surgery benefited significantly from radiotherapy (both OS and BCSS *p* < 0.001), for patients with T1N0M0 and ER-positive breast cancer (both OS and BCSS *p* < 0.001). In the subgroup of T1-2N1M0 breast cancer treated by mastectomy, patients undergoing radiotherapy had a worse survival as well (OS *p* < 0.001; BCSS *p* = 0.0907). While in the subgroup of T3N0M0 breast cancer treated by mastectomy, survival analyses showed no statistical differences between patients receiving radiation or not (OS *p* = 0.1778, BCSS *p* = 0.6957).

**Conclusions:**

This study indicated the clinical effects of radiation on older women who received different surgical treatments. Our study suggested that radiotherapy should be omitted in older women undergoing mastectomy + T3N0M0 or T1-2N1M0 and radiotherapy could be considered in women with T1N0M0 + ER-positive undergoing breast-conserving surgery.

**Supplementary Information:**

The online version contains supplementary material available at 10.1007/s12094-022-02967-9.

## Introduction

Breast cancer has the highest incidence rate among female cancers, the elderly patients accounting for 30% of all breast cancers [1]. Breast cancer in older women was generally less aggressive and more indolent than in younger women. Meanwhile, a population-based cohort study conducted that the majority of death in older patients with early breast cancer were from causes like cardiovascular and cerebrovascular diseases other than breast cancer itself [2]. Therefore, de-escalating treatments for early-stage breast cancer has always been the treatment theme for aging patients [3]. Nowadays, with the population aging and the current life expectancy of 70 year olds exceeding 15 years, it is significant to obtain absolute clinical benefits from the balance of noncancer death and overtreatment in older adults. Elder women generally have more favorable tumor biology and less advanced stage at diagnosis, but the elder woman has worse breast cancer-specific mortality. Why is that? The reasons for this, include undertreatment, inadequate data from clinical trials, and potentially age-related reduced immune surveillance [4]. At present, worse still, compared with younger patients, those older than 80 years were less likely to have a mastectomy, radiotherapy, or undergo screening for breast cancer.

Radiotherapy has already become an integral part of early-stage breast cancer treatment, including significantly controlling local tumors and improving overall survival [5]. However, adjuvant radiotherapy was also accompanied by adverse reactions such as radiation dermatitis, radiation pneumonia, limb lymphedema, heart damage, and so on. Therefore, the absolute benefit of RT was not equal for all women. Hughes [6] et al. advocated the view that the majority of deaths in older women with early breast cancer were from causes like cardiovascular and cerebrovascular diseases other than breast cancer itself and that it was necessary to stop radiating in these older women with stage I.

As for patients who underwent breast-conserving surgery, CALGB 9343 reported that there was no significant difference in the use of breast-conserving surgery plus tamoxifen or an aromatase inhibitor without breast irradiation in women with clinical stage I, ER-positive breast cancer aged 70 years or older at diagnosis in overall or disease-free survival [7, 8]. Analogous results were obtained in other studies of a similar design [9]. However, the duration of endocrine therapy for breast cancer especially those in menopausal status was still too long, which made normalization and continuity of treatment more challenged. Current evidence focuses on an inactive situation that adjuvant endocrine therapy in ER-positive breast cancer only accounts for about 49% of patients [10]. Meanwhile, compared with younger patients, those older patients were less likely to have an endocrine therapy for breast cancer [11].

In patients undergoing total breast resection, previous research showed that radiotherapy can improve overall survival (OS) and disease-free survival (DFS) rates for T3-4 or lymph node positive breast cancer [12, 13]. At the same time, an observational study indicated that women with T1-2N0 triple-negative breast cancer treated with modified radical mastectomy without radiation therapy had a significantly increased risk of locoregional recurrence compared with those treated with breast-conserving therapy [14]. In the context of increasing radiotherapy applications for patients after total mastectomy, few research has been done to address related issues in the elderly patients. Therefore, whether the radiotherapy treatment can be omitted in elder patients after mastectomy, especially in those with T1-2 or lymph node positive breast cancer was unknown.

In this study, to evaluate the effect of adjuvant radiation therapy on older women with early-stage breast cancer, we reviewed the Surveillance, Epidemiology and End Results (SEER) database of the US National Cancer Institute to compare the survival outcomes between two groups (Radiation or No Radiation). After PSM, the prognosis of patients who underwent breast-conserving surgery or mastectomy was calculated separately. The effects of radiotherapy on the survival of three special population groups (breast-conserving surgery + T1N0M0 + ER positive, mastectomy + T3N0M0, and mastectomy + T1-2N1M0) were analyzed selectively.

## Materials and methods

### Data source

The Surveillance, Epidemiology and End Results (SEER) program contains cancer incidence and mortality data from 18 population‐based registries that represent approximately 30% of the US population. We obtained data from the SEER database using the SEER*Stat software version 8.3.6, based on the November 2019 submission (1975–2017 varying).

### Patient selection

Elderly patients (≥ 70 years) with early-stage breast cancer (stage I–IIB) were identified based on the Breast-Adjusted AJCC 6th Stage. Other selection criteria included: female, diagnostic confirmation, infiltrating duct carcinoma or infiltrating lobular carcinoma, or infiltrating duct and lobular carcinoma. The exclusion criteria were as follows: (1) patients with incomplete survival data and follow‐up information; (2) patients who did not undergo surgery; (3) patients who had more than one malignancy. All patients were divided into two groups according to whether they received radiotherapy (Group1: underwent radiation, Group 2: did not undergo radiation).

### Study variables

Our main purpose was to analyze the usage of radiotherapy in elderly breast cancer patients and its impact on prognosis. Overall survival (OS) and breast cancer-specific survival (BCSS) were calculated from the date of diagnosis to the last date of available vital status. We also evaluated independent demographic and clinicopathological variables for each case, including age, year of diagnosis (before 2000, 2001–2010 and 2011–2017), histologic grade (grade 0, 1, 2, 3, 4), histologic type(ductal carcinoma, lobular carcinoma, ductal and lobular carcinoma), T stage (Breast—Adjusted AJCC 6th T), N stage (Breast—Adjusted AJCC 6th T), estrogen receptor status, HER2/neu status, molecular subtype (Her2−/ER + , Her2 + /ER + , Her2 + /ER−, and Triple Negative), type of surgery (Partial mastectomy, mastectomy), regional nodes examined number (1–5, 6–9, ≥ 10), regional nodes positive (0, 1, 2, and 3), and chemotherapy.

### Propensity score matching (PSM)

The SEER database is useful for investigating the effect of demographics, stage, surgery type, and radiation used in rare tumors or rare clinical situations. However, unlike randomized trial data, observational data regarding the efficacy of treatment versus nontreatment is confounded by selection bias. To adjust for this selection bias, Rosenbaum and Rubin proposed a propensity score matching method to estimate the average treatment effect with observational datasets.

In this study, propensity score matching (PSM) (exact match, match tolerance = 0) was performed to further evaluate the effect of radiotherapy on survival by adjusting for gender, year of diagnosis, histologic type, T stage, N stage, ER status, HER2 status, type of surgery, regional nodes positive number, and receipt of chemotherapy.

### Statistical analysis

For demographic and clinicopathological data, continuous variables such as age were compared using the *t* test or ANOVA test and categorical variables were compared using the Pearson’s chi-squared test or rank sum test. Survival curves were performed according to the Kaplan–Meier method and compared using the log-rank test. Univariate and multivariate Cox proportional hazards regression models were constructed to analyze factors associated with survival.

The prognosis of patients who underwent breast-conserving surgery or mastectomy was calculated separately. The effects of radiotherapy on the survival of three special population groups (breast-conserving surgery + T1N0M0 + ER positive, mastectomy + T3N0M0, and mastectomy + T1-2N1M0) were analyzed selectively.

Statistical significance was set at a two‐sided *p* < 0.05, and all confidence intervals (CI) are stated at the 95% confidence level. The statistical analyses were performed using SPSS statistical software (version 25.0, IBM Corp, Armonk, NY) and Stata statistical software (version 16.0, Stata Corp LLC, College Station, Texas).

## Results

Totally, 106,553 older women (≥ 70 years) with early-stage (stage I–IIB) breast cancer were identified. 48,630 patients had received radiotherapy (Group1), while 57,923 patients had not (Group2). The demographics and clinicopathological characteristics of the three groups are summarized in Table 1.

**Table 1:** Patient characteristics

Clinical characteristics	No. of patients (%)		*P*
	Radiation *n* = 48,630	No radiation *n* = 57,923	
Age at diagnosis: Mean ± SD, y	75.77 ± 4.60	77.55 ± 5.56	< 0.001
Year of diagnosis			< 0.001
1988–2000	7455 (15.33%)	14,544 (25.11%)	
2001–2010	24,011 (49.37%)	27,148 (46.87%)	
2011–2017	17,164 (35.30%)	16,231 (28.02%)	
Tumor grade			< 0.001
Unknown	2326 (4.78%)	5269 (9.10%)	
Grade I	12,608 (25.93%)	12,226 (21.11%)	
Grade II	22,658 (46.59%)	25,234 (43.56%)	
Grade III	10,817 (22.24%)	14,798 (25.55%)	
Grade IV	221 (0.45%)	396 (0.68%)	
Histologic type			0.003
Ductal carcinoma	39,657 (81.55%)	46,794 (80.79%)	
Lobular carcinoma	5323 (10.95%)	6695 (11.56%)	
Ductal and lobular carcinoma	3650 (7.51%)	4434 (7.65%)	
T			< 0.001
T0	7 (0.01%)	3 (0.01%)	
T1	37,784 (77.70%)	37,936 (65.49%)	
T2	10,221(21.02%)	18,953(32.72%)	
T3	618(1.27%)	1,031(1.78%)	
N			< 0.001
N0	39,731 (81.70%)	45,221 (78.07%)	
N1	8,899 (18.30%)	12,702 (21.93%)	
Stage			< 0.001
I	32,595 (67.03%)	32,111 (55.44%)	
IIA	11,714 (24.09%)	17,907 (30.92%)	
IIB	4321 (8.89%)	7905 (13.65%)	
ER status			< 0.001
Unknown	2467 (5.07%)	7552 (13.04%)	
Positive	40,630 (83.55%)	42,724 (73.76%)	
Negative	5533 (11.38%)	7647 (13.20%)	
HER2 status^a^			< 0.001
Unknown	29,245 (60.14%)	40,013 (69.08%)	
Positive	1703 (3.50%)	2117 (3.65%)	
Negative	17,682 (36.36%)	15,793 (27.27%)	
Molecular subtype^a^			< 0.001
Unknown	29,254 (60.16%)	40,044 (69.13%)	
HR + /Her2−	16,103 (33.11%)	14,138 (24.41%)	
HR + /Her2 +	1279 (2.63%)	1504 (2.60%)	
HR-/Her2 +	424 (0.87%)	608 (1.05%)	
HR−/Her2−	1570 (3.23%)	1629 (2.81%)	
Surgery of breast			< 0.001
Partial mastectomy	45,473 (93.51%)	17,557 (30.31%)	
Mastectomy	3157 (6.49%)	40,366 (69.69%)	
Regional nodes positive			< 0.001
0	39,813(81.87%)	45,292(78.19%)	
1	5726 (11.77%)	7730 (13.35%)	
2	2045 (4.21%)	3224 (5.57%)	
3	1046 (2.15%)	1677 (2.90%)	
Chemotherapy			< 0.001
Yes	6547 (13.46%)	5918 (10.22%)	
No/unknown	42,083 (86.54%)	52,005 (89.78%)	

### Application of radiotherapy in elderly patients with early-stage breast cancer

Overall, 45.64% of elderly patients had been treated with radiation. The application of radiotherapy increased with the years, with 33.76% in 1988–2000, 46.65% in 2001–2010 and 51.11% in 2011–2017. However, the use of radiotherapy in elderly patients undergoing breast-conserving surgery was gradually decreasing (77.71% in 1988–2000, 72.67% in 2001–2010 and 69.25% in 2011–2017), especially in patients with T1N0M0 and ER-positive breast cancer (82.41% in 1988–2000, 74.19% in 2001–2010 and 67.57% in 2011–2017). Radiotherapy was less used in elderly patients receiving mastectomy but there was an increasing trend (4.58% in 1988–2000, 7.49% in 2001–2010, and 10.79% in 2011–2017), especially in patients with T3 or N1 breast cancer (Supplementary Material, Table 1)

### Survival analyses

**Fig. 1:**
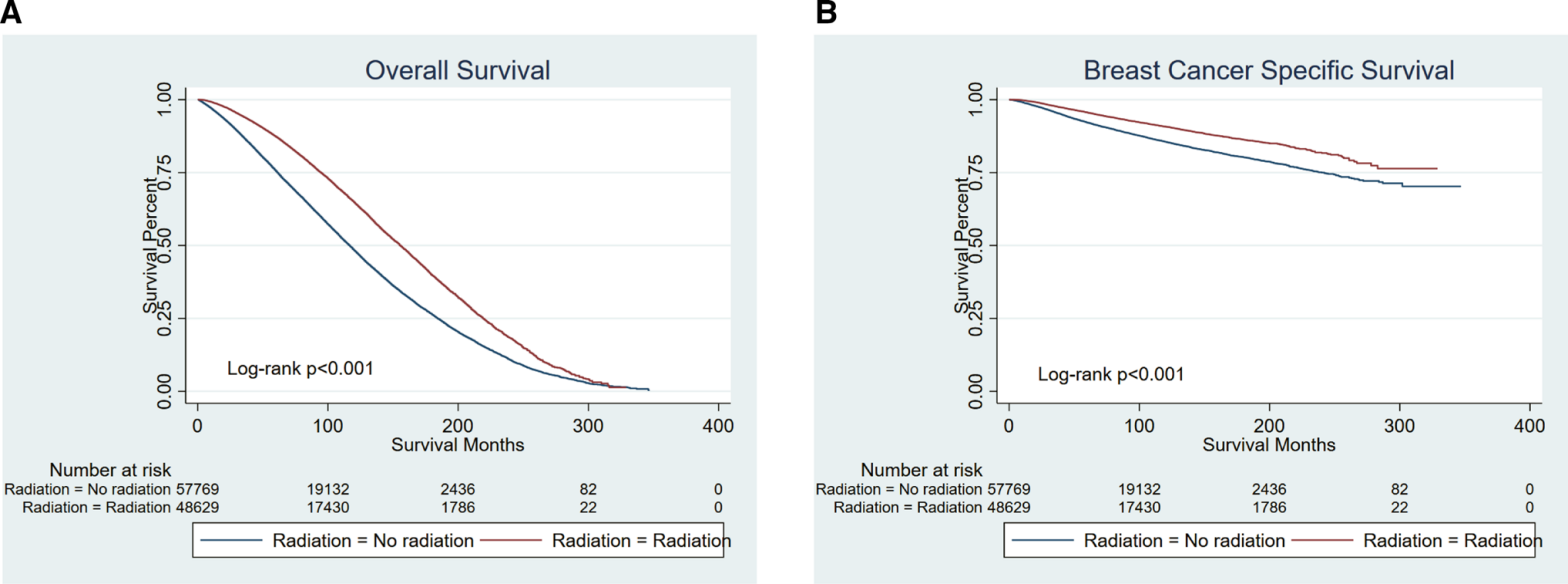
Kaplan–Meier curves of all elderly patients with early-stage breast cancer (before PSM). Patients who had been treated by radiotherapy had a better survival (both OS and BCSS *p* < 0.001). **A** OS, **B** BCSS

The median length of follow-up was 76 months for the radiation group, and 70 months for the no radiation group (*p* < 0.001). Multivariate Cox proportional hazards regression analyses showed that radiation was an independent risk factor both in the OS and BCSS (Supplementary Material, Table 2, Table 3). Kaplan–Meier curves comparing survival times between two groups are presented in Fig. 1. In summary, patients who had been treated by radiotherapy (Group1) had a better survival (both OS and BCSS, *p* < 0.001).

### Propensity score matching (PSM)

Propensity score matching (PSM) was performed to further evaluate the effect of radiotherapy on survival by adjusting for age, year of diagnosis, grade, histologic type, T stage, N stage, ER status, HER2 status, type of surgery, and receipt of chemotherapy. After PSM, 19,372 patients were included in each group and all critical variables were balanced (Table 2). In each group, there were 16,367 patients undergoing breast-conserving surgery and 3005 patients undergoing mastectomy.

**Table 2:** Patient characteristics after PSM

Clinical characteristics	No. of patients (%)		*P*
	Radiation *n* = 19,372	No radiation *n* = 19,372	
Age at diagnosis: Mean ± SD, y	76.78 ± 5.25	77.17 ± 5.37	< 0.001
Year of diagnosis			1
1988–2000	2361 (12.19%)	2361 (12.19%)	
2001–2010	9424 (48.65%)	9424 (48.65%)	
2011–2017	7587 (39.16%)	7587 (39.16%)	
Tumor grade			1
Unknown	918 (4.74%)	918(4.74%)	
Grade I	5080 (26.22%)	5080(26.22%)	
Grade II	8808 (45.47%)	8808(45.47%)	
Grade III	4504 (23.25%)	4504 (23.25%)	
Grade IV	62 (0.32%)	62 (0.32%)	
Histologic type			1
Ductal carcinoma	16,007 (82.63%)	16,007 (82.63%)	
Lobular carcinoma	2023 (10.44%)	2023 (10.44%)	
Ductal and lobular carcinoma	1342 (6.93%)	1342 (6.93%)	
T			1
T0	1 (0.01%)	1 (0.01%)	
T1	14,131 (72.95%)	14,131 (72.95%)	
T2	4882 (25.20%)	4882 (25.20%)	
T3	358 (1.85%)	358 (1.85%)	
N			1
N0	15,444 (79.72%)	15,444 (79.72%)	
N1	3928 (20.28%)	3928 (20.28%)	
Stage			1
I	12,335 (63.67%)	12,335 (63.67%)	
IIA	4548 (23.48%)	4548 (23.48%)	
IIB	2489 (12.85%)	2489 (12.85%)	
ER status			1
Unknown	1363 (7.04%)	1363 (7.04%)	
Positive	15,818 (81.65%)	15,818 (81.65%)	
Negative	2191 (11.31%)	2191 (11.31%)	
HER2 status^a^			1
Unknown	11,118 (57.39%)	11,118 (57.39%)	
Positive	754 (3.89%)	754 (3.89%)	
Negative	7500 (38.72%)	7500 (38.72%)	
Molecular subtype^a^			1
Unknown	11,121 (57.41%)	11,121 (57.41%)	
HR + /Her2−	6892 (35.58%)	6890 (35.57%)	
HR + /Her2 +	562 (2.90%)	564 (2.91%)	
HR−Her2 +	192 (0.99%)	190 (0.98%)	
HR−/Her2−	605 (3.12%)	607 (3.13%)	
Surgery of breast			1
Partial mastectomy	16,367 (84.49%)	16,367 (84.49%)	
Mastectomy	3005 (15.51%)	3005 (15.51%)	
Regional nodes positive			< 0.001
0	15,469 (79.85%)	15,485 (79.93%)	
1	2519 (13.00%)	2312 (11.93%)	
2	945 (4.88%)	992 (5.12%)	
3	439 (2.27%)	583 (3.01%)	
Chemotherapy			1
Yes	2579 (13.31%)	2579 (13.31%)	
No/unknown	16,793 (86.69%)	16,793 (86.69%)	

### Survival analyses after PSM

**Fig. 2:**
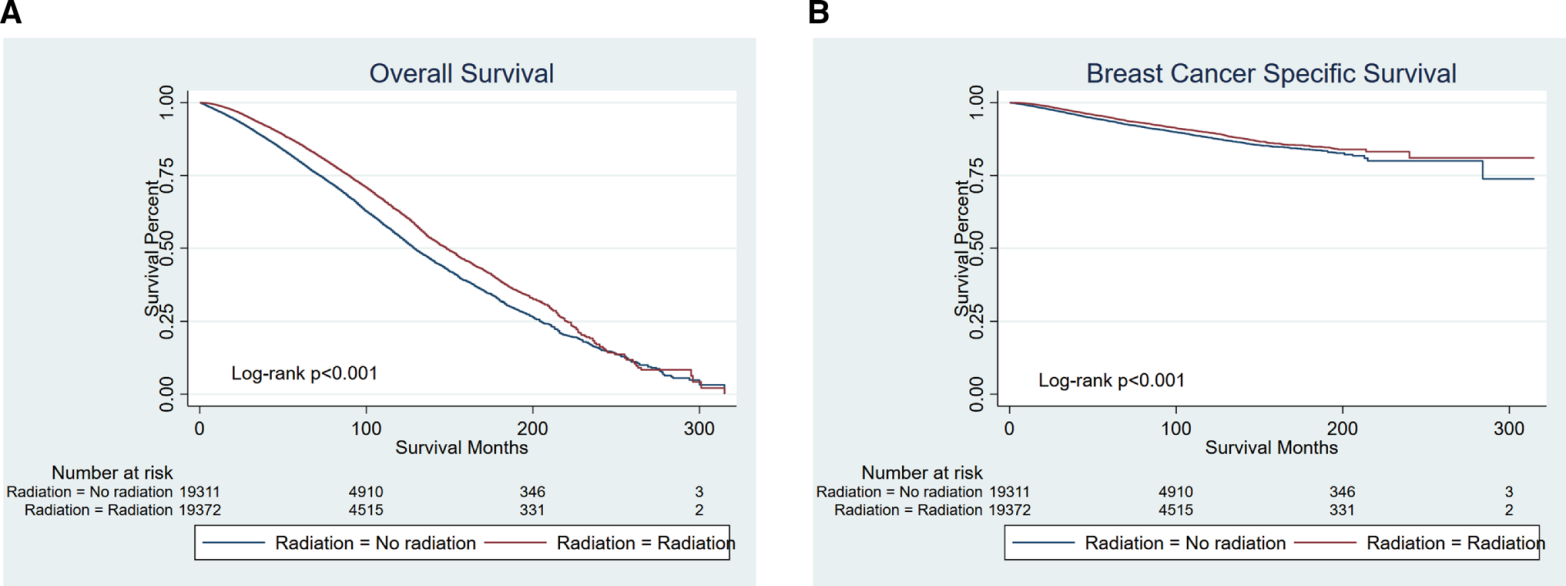
Kaplan–Meier curves of all elderly patients with early-stage breast cancer (after PSM). Patients who had been treated by radiotherapy had a better survival (both OS and BCSS *p* < 0.001). **A** OS, **B** BCSS

Kaplan–Meier curves comparing survival time between the radiation group and no radiation group are presented in Fig. 2. After PSM, patients undergoing radiotherapy (Group1) had a better survival as well (both OS and BCSS *p* < 0.001). Separately, the effect of radiotherapy on the prognosis of patients undergoing breast-conserving surgery or mastectomy is completely opposite.

### Effect of radiotherapy on the survival prognosis of older women undergoing breast-conserving surgery

**Fig. 3:**
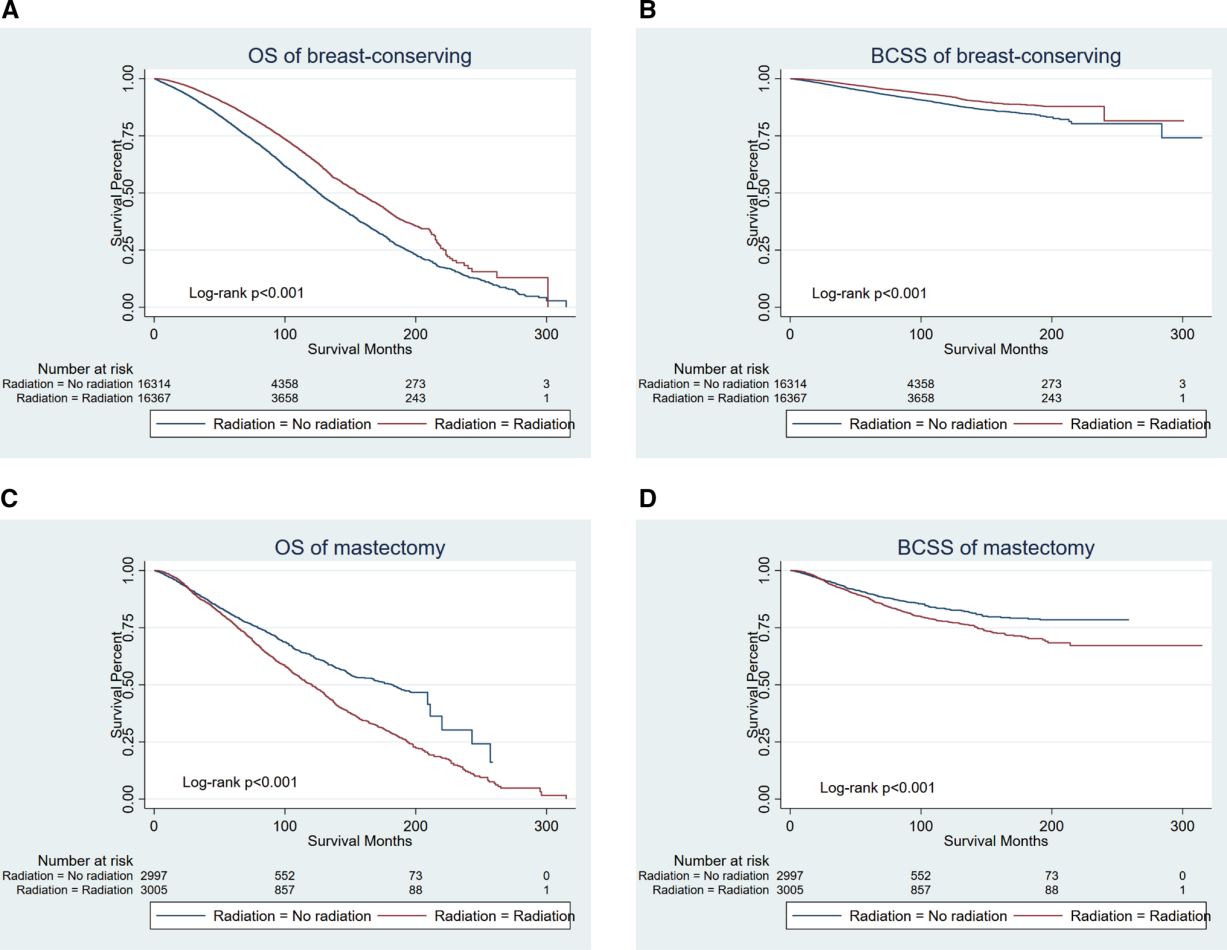
Kaplan–Meier curves of patients according to breast surgery (after PSM). Patients undergoing breast-conserving surgery benefited significantly from radiotherapy (both OS and BCSS *p* < 0.001). For patients treated with mastectomy, radiation led to worse outcomes (both OS and BCSS *p* < 0.001). **A** Overall survival of patients undergoing breast-conserving surgery; **B** breast cancer-specific survival of patients undergoing breast-conserving surgery; **C** overall survival of patients undergoing mastectomy; **D** breast cancer-specific survival of patients undergoing mastectomy

Patients undergoing breast-conserving surgery benefited significantly from radiotherapy (Fig. 3A, B) (both OS and BCSS *p* < 0.001). Our subgroup analysis of BCSS indicated that several subgroups that underwent breast-conserving surgery had different effects after radiotherapy, regarding with year of diagnosis, chemotherapy, T staging, the number of positive lymph nodes, the number of axillary lymph nodes removed, and molecular subtype (Supplementary Material, fig. 1)

**Fig. 4:**
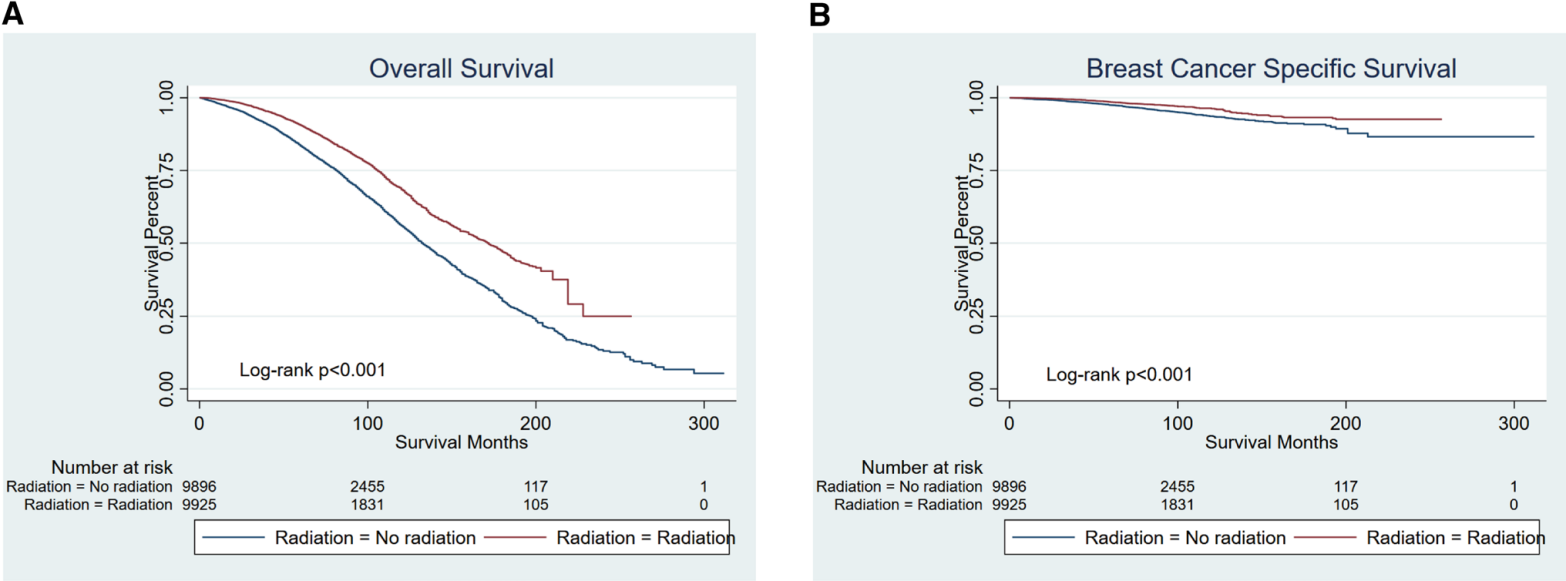
Kaplan–Meier curves of patients with T1N0M0 and ER-positive breast cancer who had been treated with breast-conserving surgery for older women with T1N0M0 and ER-positive breast cancer who had been treated with breast-conserving surgery, patients in radiotherapy group had a better prognosis (both OS and BCSS *p* < 0.001). **A** OS, **B** BCSS

For older women with T1N0M0 and ER-positive breast cancer who had been treated with breast-conserving surgery, there were 9925 patients in each group after PSM. Survival analyses showed that patients in the radiotherapy group had a better prognosis (Fig. 4A, B) (both OS and BCSS, *p* < 0.001).

### Effect of radiotherapy on the survival prognosis of older women undergoing mastectomy

No matter OS or BCSS, radiation led to worse survival in patients who had received mastectomy (Fig. 3C, B) (both OS and BCSS *p* < 0.001). The following subgroup analysis of BCSS demonstrated that age, T staging, number of positive lymph nodes, and molecular subtype were significantly correlated with the clinical benefit of radiotherapy for patients with mastectomy (Supplementary Material, fig. 2).

**Fig. 5:**
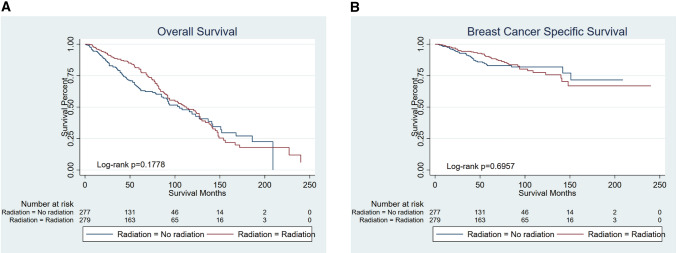
Kaplan–Meier curves of patients with T3N0M0 breast cancer and underwent mastectomy. No statistical differences between patients treated with radiation or not (OS *p* = 0.1778; BCSS *p* = 0.6957). **A** OS, **B** BCSS

After PSM, there were only 279 patients in each group who had T3N0M0 breast cancer and underwent a mastectomy. Survival analyses showed no statistical differences between patients treated with radiation or not (Fig. 5A, B) (OS *p* = 0.1778) (BCSS *p* = 0.6957).

**Fig. 6:**
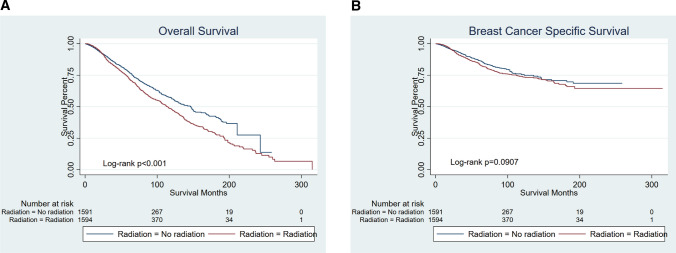
Kaplan–Meier curves of patients with T1-2N1M0 breast cancer and underwent mastectomy. Patients undergoing radiotherapy had a worse survival (OS *p* < 0.001; BCSS *p* = 0.0907). **A** OS, **B** BCSS

After PSM, there were 1594 patients in each group who had T1-2N1M0 breast cancer and underwent a mastectomy. Kaplan–Meier curves comparing survival time between the radiation group and no radiation group are presented in Fig. 6. Surprisingly patients undergoing radiotherapy had a worse survival (OS *p* < 0.001; BCSS *p* = 0.0907).

## Discussion

Surgery is the principal and effective treatment for breast cancer, but it is usually unavoidable for residual disease in the form of scattered micrometeorite tumor cells. Radiation therapy has been shown to reduce the risk of breast cancer mortality as well as locoregional recurrence [15]. Previous studies have confirmed that patients with high-risk tumors (> 5 cm) [16] or patients with four or more positive lymph nodes can benefit from radiotherapy [17]. However, the role of post-mastectomy radiotherapy (PMRT) in women patients with pathologic N1 breast cancer, remains argumentative [18]. Due to the special biology of breast cancer in this age group, few trials included elderly patients older than 70 years, let alone eligible older patients. Our study tends to uncover that aging woman should be assessed individually, with different pathological features, surgical comorbidity, and patient expectancy as determining factors in an assessment of the risks and benefits of radiation.

In this study, we identified 10,415 women with early-stage breast cancer and divided them into three special subgroups (breast-conserving surgery + T1N0M0 + ER-positive, mastectomy + T3N0M0, and mastectomy + T1-2N1M0, respectively) based on the guideline classification and the indications for surgery. Elderly breast cancer patients tend to choose modified radical mastectomy and breast-conserving surgery with radiotherapy [19, 20]. However, when accompanied by multicentric disease, chest wall involvement, special pathological tissue types (Paget disease), or high-risk factors, patients with mastectomy have greater clinical benefits than those with BCS. Different breast surgery with or without irradiation in elderly women with early breast cancer was still worthy of attention. Importantly, our study found that the rate of breast cancer radiotherapy was increasing regardless of the elderly or the young, but the radiotherapy of the elderly breast-conserving (BCS) patients was indeed gradually decreasing, especially in the patients with T1N0M0 and hormone receptor-positive tumors. Maybe the downward tendency of radiotherapy after BCS was referenced to the results of the above cohort studies (CALGB 9343 [7] and PRIME II [9]), which suggested that those special subgroups in whom irradiation may not provide meaningful overall benefits. As such, National Comprehensive Cancer Network breast cancer guidelines were changed to allow the omission of radiotherapy in older patients with hormone receptor-positive cancer after breast-conserving surgery. Conversely, after PSM, our study has shown that those elderly subgroups with T1N0M0 and ER positive who underwent radiotherapy had a better clinical prognosis in OS and BCSS. Some previous studies indicated that the transcriptional program of ER genome drive tumor cell metastasis and proliferation [21] [22]. It was a bit different from our conventional knowledge of tumor radiotherapy. The reason for this contradiction in the prospective randomized study we guess is as follows. First, approximately 70% of breast cancer patients with ER + are candidates for endocrine treatment [23], especially in postmenopausal women [24]. However, due to the unsatisfactory duration of endocrine therapy and treatment compliance, it is bound inevitably to an adverse prognosis, especially in early-stage breast cancer patients who are omitted for radiotherapy. Meanwhile, previous studies have demonstrated that less than 50% of patients have completed standard endocrine therapy. Second, the PRIME II study [9] required that histologically graded 3 and vascular tumor thrombi simultaneously were excluded, whereas our data cannot make such a restriction. Therefore, selection bias was available in this study, because this part of eligible patients with histologically graded 3 and vascular tumor thrombi may gain more absolute benefit from radiotherapy. Furthermore, the CALGB 9343 trials failed to eliminate the confounding bias of Tam’s endocrine therapy and selected patients were more likely to die of complications rather than breast cancer itself, which may cause no statistical difference in OS or BCSS. In fact, an analysis of Medicare data in the USA showed that the CALGB trial findings had only reduced the use of adjuvant radiotherapy by 3% [7]. Based on its low effect in actual clinical use, we had reason to believe that the omission of radiotherapy was still contradictor in T1N0M0 patients aged 70 years or older with ER + after BCS treatment. Therefore, our results suggested that the elderly female patients staged earlier after experiencing BCT should need more adequately radiotherapy rather than giving up radiotherapy in terms of survival rate, especially in patients with histological grade, vascular tumor thrombus, and other high-risk factors.

T3N0M0 [T3: Tumor > 5 cm in greatest dimension without regional lymph node metastases or distant metastases] breast cancer represents a rare disease, occurring in approximately 2% of all breast cancers [25]. National Surgical Adjuvant Breast and Bowel Project (NSABP) randomized trials [26] reviewing 313 women with T3N0M0 disease had only a 7% absolute risk of isolated locoregional recurrence and had a point of view that female patients cannot benefit much from PMRT, aggravating their damage. Consistent with the above results, our finding showed that the use of PMRT adds no significant benefit in terms of OS or BCSS (OS *p* = 0.1778; BCSS *p* = 0.6957). Therefore, PMRT was not recommended in selected T3N0M0 patients. Moreover, the use of radiotherapy in elderly patients undergoing total mastectomy increased over the years. However, T1-2N1M0 breast cancer patients after radiotherapy have a more adverse prognosis in our study. Previously, our group published a population-based study of 45,646 patients from SEER, demonstrating that PMRT did not improve the breast cancer-specific survival (BCSS) in patients with stage T1-2N1M0 [18]. Herein, considering the adverse effects of RT, for elder patients whose physical conditions were worse than that of young women, PMRT may be dispensable [27] [28].Therefore, elderly female patients in T1-2N1M0 might not benefit from radiation therapy and the aging group can be exempted from radiotherapy.

We acknowledge the limitations of the present study. First, radiotherapy has a major impact on reducing the risk of local recurrence, especially in patients with breast-conserving surgery for early-stage breast cancer [29]. However, our article solely mentioned the effects of radiotherapy on the survival prognosis (both OS and BCSS), because the data about local tumor control was not included in the SEER database. Second, the limitations of our study were the absence of detailed information on endocrine treatment, which may easily affect the decision of female patients with early breast cancer. Third, due to the underrepresentation of older patients [30], the sample of elderly patients undergoing mastectomy was relatively scarce. So, it is worth considering whether a consequence of selection bias or an independent effect in the study. At last, our study has the usual limitations of descriptive epidemiology which are retrospective registry assessment, missing data in SEER, no standardized definitions, and lack of individual-level risk factor data.

## Conclusion

In conclusion, our study suggested that radiotherapy should be omitted in older women undergoing mastectomy + T3N0M0 or T1-2N1M0 and radiotherapy could be considered in elder women with T1N0M0 + ER positive undergoing breast-conserving surgery. Further studies were warranted to combine survival outcomes, local control effects, and adverse reactions to investigate the absolute clinical benefit of adjuvant radiotherapy in elderly women.

## Supplementary Information

Below is the link to the electronic supplementary material.

Supplementary file1 (DOCX 691 KB)

## Data Availability

National Cancer Institute. Surveillance, Epidemiology and End Results (SEER) Program (http://www.seer.cancer.gov) SEER*Stat Database: Incidence–SEER 18 Regs Custom Data (with additional treatment fields), Nov 2018 Sub (1975–2016 varying)–Linked To County Attributes–Total US, National Cancer Institute, DCCPS, Surveillance Research Program, released April 2019, based on the November 2018 submission. Available at: https://www.seer.cancer.gov/data/, Accessed November 19, 2019.
