# Supplementary material for: Adjuvant radiation therapy for older women with early-stage breast cancer: a propensity-matched SEER analysis
Source: Clin Transl Oncol. 2022 Oct 13;25(2):523–34. doi: 10.1007/s12094-022-02967-9 (PMC9873780; doi:10.1007/s12094-022-02967-9)
Supplement: Supplementary file 1 — Supplementary file1 (DOCX 691 KB) [file 12094_2022_2967_MOESM1_ESM.docx]

| **Supplementary Table1** | | | |
| --- | --- | --- | --- |
|  |  | ≥70岁 | <70岁 |
| Group | Year of diagnosis | Radiation | Radiation |
| Total | 1988-2000 | 7495(33.76%) | 28667(46.59%) |
|  | 2001-2010 | 24085(46.65%) | 94082(54.85%) |
|  | 2011-2017 | 17230(51.11%) | 66793(57.74%) |
| Breast conserving surgery | 1988-2000 | 6873(77.71%) | 25799(80.46%) |
|  | 2001-2010 | 22534(72.67%) | 83997(78.36%) |
|  | 2011-2017 | 16099(69.25%) | 58589(81.67%) |
| Mastectomy | 1988-2000 | 610(4.58%) | 2831(9.64%) |
|  | 2001-2010 | 1542(7.49%) | 10036(15.63%) |
|  | 2011-2017 | 1128(10.79%) | 8180(18.64%) |
| Breast conserving surgery+  T1N0M0,  ER+ | 1988-2000 | 3548(81.41%) | 11322(86.31%) |
|  | 2001-2010 | 13665(74.19%) | 44902(84.03%) |
|  | 2011-2017 | 9954(67.57%) | 34923(86.10%) |
| Mastectomy+  T3 | 1988-2000 | 46(18.18%) | 188(28.19%) |
|  | 2001-2010 | 168(29.42%) | 901(49.29%) |
|  | 2011-2017 | 132(34.29%) | 747(51.38%) |
| Mastectomy +N1 | 1988-2000 | 267(8.19%) | 1508(16.38%) |
|  | 2001-2010 | 786(13.40%) | 6079(27.28%) |
|  | 2011-2017 | 685(23.92%) | 5529(39.94%) |
| Mastectomy _+_T1-2N0 | 1988-2000 | 297(3.03%) | 1135(5.82%) |
|  | 2001-2010 | 588(4.15%) | 3056(7.62%) |
|  | 2011-2017 | 311(4.31%) | 1904(6.66%) |

| **Supplementary Table 2.** **Multivariate Cox proportional hazards regression analyses of**  **Overall Survival** | | | | |
| --- | --- | --- | --- | --- |
| **Clinical characteristics** | ***P*** | **Hazard ratio** | **95% Confidence Interval** | |
| Year of diagnosis | ＜0.001 | 0.939 | 0.918 | 0.959 |
| Grade | ＜0.001 | 1.085 | 1.074 | 1.096 |
| Histologic type | ＜0.001 | 0.947 | 0.932 | 0.962 |
| T | ＜0.001 | 1.437 | 1.410 | 1.464 |
| N | ＜0.001 | 1.152 | 1.102 | 1.205 |
| Radiation | ＜0.001 | 0.683 | 0.666 | 0.702 |
| Chemotherapy | ＜0.001 | 0.621 | 0.599 | 0.643 |
| Surgery | 0.001 | 1.045 | 1.018 | 1.073 |
| Nodesexamined | ＜0.001 | 0.977 | 0.965 | 0.989 |
| Regionalnodespositive1988 | ＜0.001 | 1.118 | 1.090 | 1.147 |
| ER | ＜0.001 | 1.078 | 1.058 | 1.099 |

| **Supplementary Table 3.** **Multivariate Cox proportional hazards regression analyses of**  **Breast Cancer Specific Survival** | | | | |
| --- | --- | --- | --- | --- |
| **Clinical characteristics** | ***P*** | **Hazard ratio** | **95% Confidence Interval** | |
| Year of diagnosis | ＜0.001 | 0.821 | 0.785 | 0.859 |
| Grade | ＜0.001 | 1.316 | 1.284 | 1.349 |
| Histologic type | ＜0.001 | 0.925 | 0.892 | 0.959 |
| T | ＜0.001 | 2.059 | 1.982 | 2.139 |
| N | ＜0.001 | 1.521 | 1.402 | 1.650 |
| Radiation | ＜0.001 | 0.779 | 0.736 | 0.824 |
| Chemotherapy | 0.001 | 0.901 | 0.850 | 0.956 |
| Surgery | ＜0.001 | 1.136 | 1.074 | 1.203 |
| Node-sexamined | 0.134 | 0.980 | 0.954 | 1.006 |
| Nodes-positive | ＜0.001 | 1.232 | 1.180 | 1.285 |
| ER | ＜0.001 | 1.333 | 1.280 | 1.389 |

| 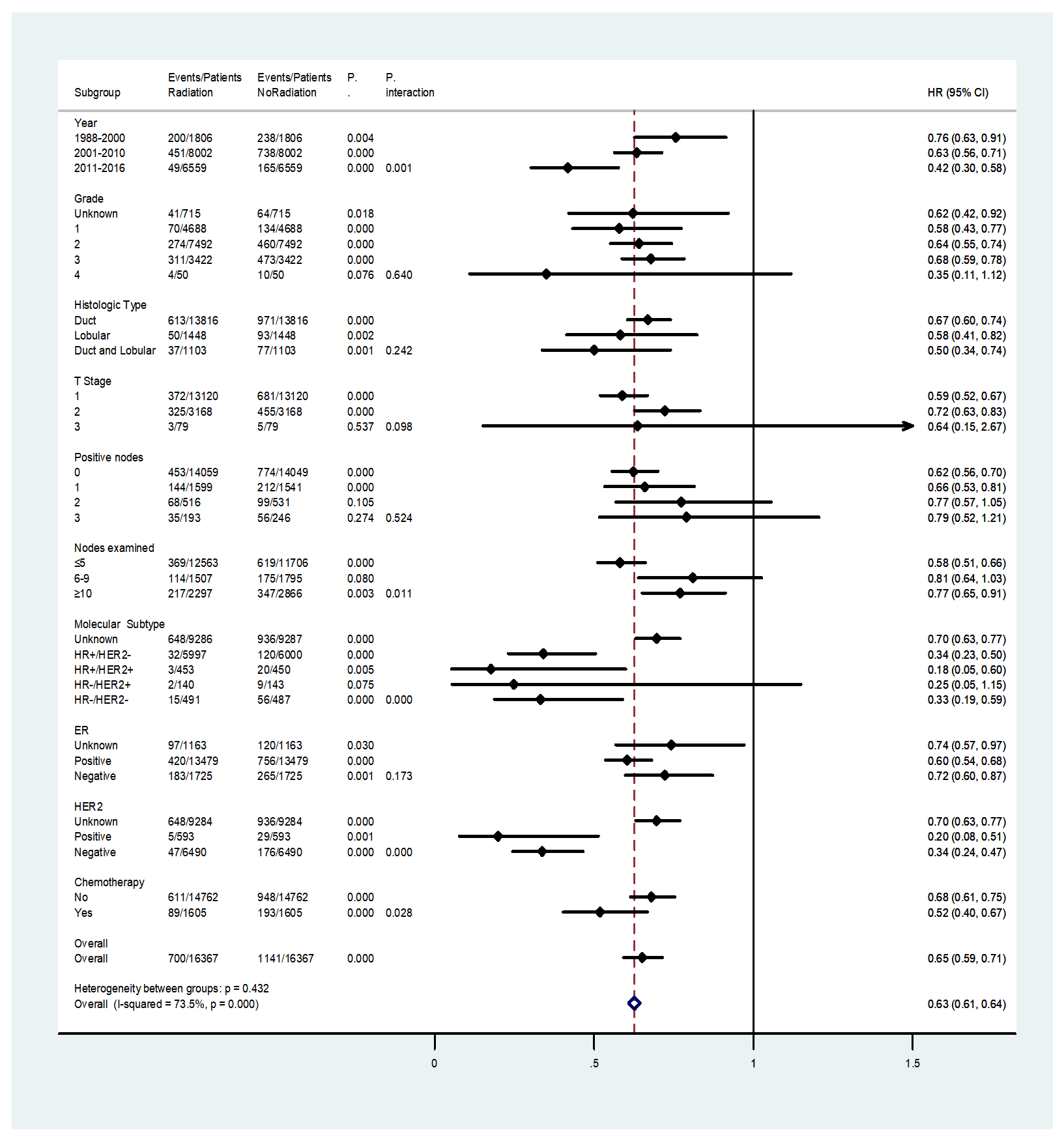 |
| --- |
| **Supplementary Figure 1. Subgroup analyses of BCSS for patients undergoing breast conserving surgery**  For patients with breast conserving surgery, year of diagnosis, chemotherapy, T staging, the number of positive lymph nodes, the number of axillary lymph nodes removed and molecular subtype were significantly correlated with the survival benefit of radiotherapy. |

| 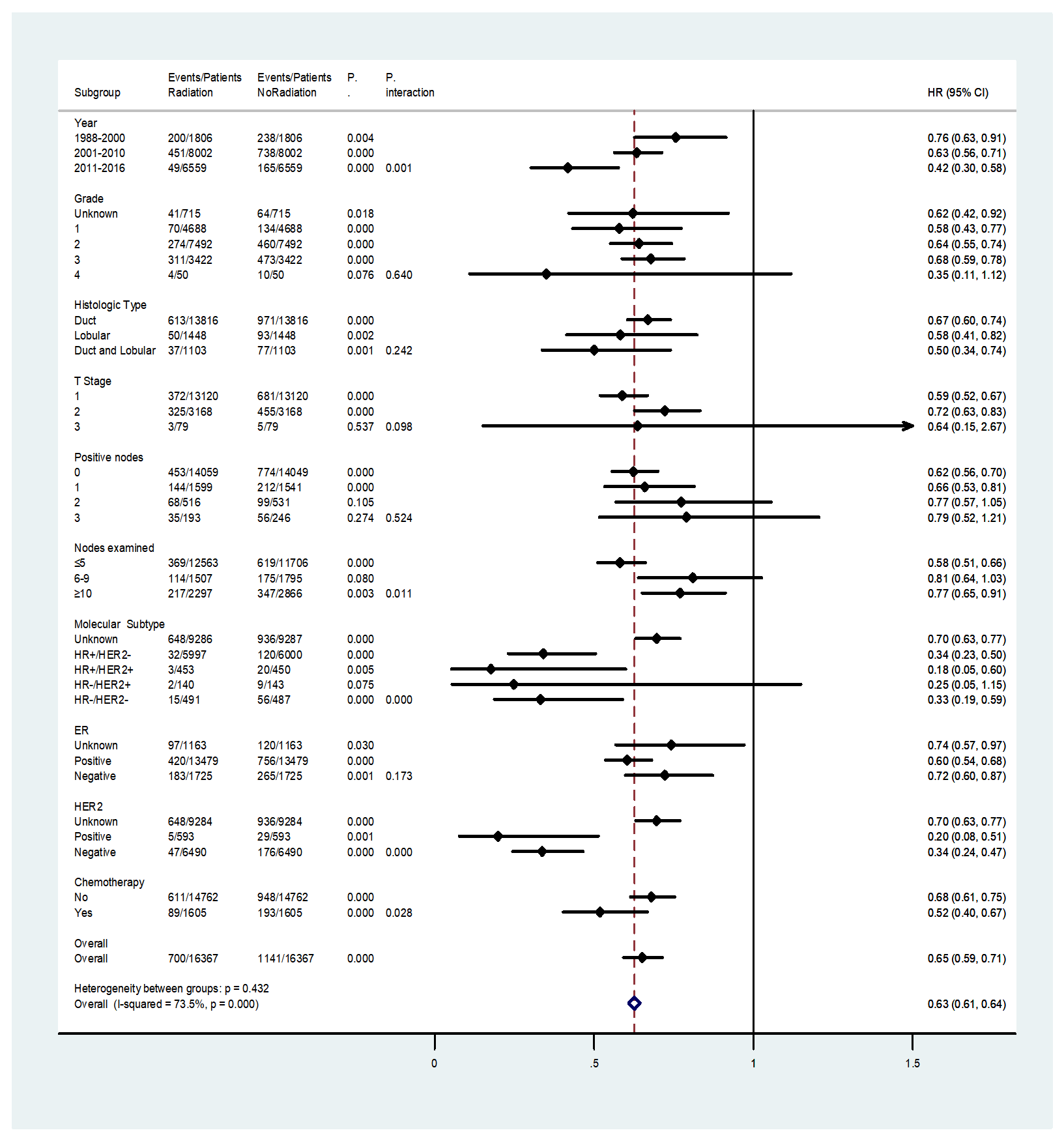 |
| --- |
| **Supplementary Figure 2. Subgroup analyses of BCSS for patients undergoing mastectomy**  For patients with mastectomy, age, T staging, number of positive lymph nodes, and molecular subtype were only significantly correlated with the clinical benefit of radiotherapy |
